# Discovery and biological evaluation of novel *N*-aryl-*N*′-methylbenzodrazides against *Schistosoma japonicum in vitro* and *in vivo*

**DOI:** 10.1128/spectrum.01932-25

**Published:** 2025-12-16

**Authors:** Lvyin Zheng, Yumei Zhong, Yinyin Li, Peiyi Chen, Nina Cheng, Xiaofeng Hua, Lu Ouyang, Nianhua Luo, Jiayi Shen, Yongdong Li, Wei Guo

**Affiliations:** 1 Jiangxi Provincial Key Laboratory of Synthetic Pharmaceutical Chemistry, Gannan Normal University 12450, Ganzhou, Jiangxi Province, People's Republic of China; 2 School of Pharmacy, Gannan Medical University 74554, Ganzhou, Jiangxi Province, People's Republic of China; University of Illinois Urbana-Champaign, Urbana, Illinois, USA

**Keywords:** *N*-aryl-*N*′-methylbenzohydrazides, antischistosomal activity, structure-activity relationship, toxicity, mouse model

## Abstract

Millions of people in tropical and subtropical regions rely on praziquantel to treat schistosomiasis. The limitations of current treatments and the emerging risk of praziquantel resistance highlight an urgent need for new chemotherapeutic agents. Here, we report the discovery of a novel series of *N*-aryl-*N*′-methylbenzohydrazides exhibiting potent activity against *Schistosoma japonicum* (Chinese strain). Among these, compound 41 demonstrated potent *in vitro* activity against adult *S. japonicum*, with an LC_50_ (72 h) value of 61.40 ± 1.80 μM. Ultrastructural analysis revealed that this lead compound induced tegumental disruption mechanistically comparable to praziquantel, manifested through syncytial layer disintegration, subtegumental musculature exposure, and pronounced contraction of both oral and ventral suckers. In murine schistosomiasis models, oral administration of compound 18 achieved a significant 40% worm burden reduction against juvenile-stage worms, while compound 5 demonstrated a 34% reduction against adult-stage worms. *In vivo* therapeutic assessments confirmed that compounds 5, 18, and 41 ameliorated hepatic pathology. Notably, mice administered compound 18 during juvenile worm development displayed significantly reduced egg burdens. Toxicological profiling indicated transient nephrotoxicity associated with compound 18 during juvenile-stage treatment, whereas compounds 5 and 41 exhibited favorable safety profiles across all developmental stages without observable nephrotoxicity. Acute oral toxicity studies confirmed low acute toxicity for all three candidates. The combined schistosomicidal efficacy, organ-protective effects, and superior safety profile of compound 5 position it as a highly promising lead candidate for further preclinical development. This chemotype provides a robust foundation for novel antischistosomal drug discovery in the post-praziquantel era.

**IMPORTANCE:**

With praziquantel (PZQ) as the sole therapeutic option for schistosomiasis, emerging drug resistance underscores the critical need for new agents. We identified a novel series of *N*-aryl-*N*′-methylbenzohydrazides with potent activity against *Schistosoma japonicum*. Lead compound 41 showed significant *in vitro* efficacy (LC_50_ = 61.40 ± 1.80 μM) and induced tegumental disruption similar to PZQ. In murine models, compounds 18 and 5 reduced juvenile and adult worm burdens by 40% and 34%, respectively, and alleviated hepatic pathology. Compound 5 exhibited a favorable safety profile without nephrotoxicity. With its potent schistosomicidal activity, organ protection, and low toxicity, compound 5 represents a promising lead compound for preclinical development, offering a new chemotype for post-PZQ drug discovery.

## INTRODUCTION

Schistosomiasis, a neglected tropical disease of major public health concern, is caused by parasitic trematodes of the genus *Schistosoma* (1–3). Ranked as the second most devastating parasitic disease globally after malaria, this helminthiasis is endemic in 78 tropical and subtropical countries, afflicting approximately 200 million people worldwide (4). The schistosome lifecycle comprises six distinct developmental stages: adult worms, eggs, miracidia, sporocysts, cercariae, and schistosomula (5). Optimal disease containment requires a multimodal strategy integrating three interventions: mass drug administration of schistosomicidal agents, targeted molluscicide application to control intermediate snail host populations, and ecological management coupled with biological containment to disrupt transmission (6–10). Chemotherapy remains a critical intervention for controlling schistosomiasis spread (11). Current therapeutic approaches predominantly rely on monotherapy with praziquantel (PZQ), the sole WHO-recommended antischistosomal agent (12). However, extensive dependence on this single chemotherapeutic raises significant concerns regarding the potential emergence of praziquantel-resistant strains (13, 14). Pharmacologically, PZQ exhibits potent activity against adult worms but demonstrates minimal efficacy against juvenile stages (15). This therapeutic gap, coupled with persistent drug resistance threats, underscores the urgent need for innovative anthelmintics to ensure sustainable schistosomiasis control.

**Fig 1:**
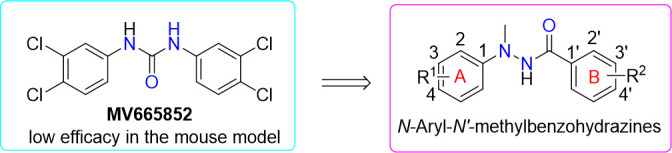
Background for a design of the target compounds.

Analogous to Plasmodium spp., schistosomes utilize host-derived heme as an essential metabolic cofactor during blood-feeding (16). This metabolic parallelism has prompted researchers to explore drug repurposing by screening established antimalarials for cross-phylum anthelmintic activity (17). Notably, pharmacological agents with dual antiparasitic effects have been identified, including artemisinin derivatives, mefloquine, and quinine-based compounds, which exhibit dose-dependent antischistosomal efficacy in both *in vitro* and *in vivo* models (18–20). In a seminal proof-of-concept study, Keiser et al. conducted a high-throughput phenotypic screen of the Medicines for Malaria Venture (MMV) Malaria Box library, a curated collection of 400 pharmacologically active compounds with confirmed antiplasmodial properties (21, 22). This effort identified MMV665852 as the most promising chemotype, demonstrating broad-spectrum schistosomicidal activity *in vitro* against both juvenile and adult *Schistosoma mansoni* (Fig. 1) (23). However, *in vivo* assessment revealed minimal antischistosomal efficacy, evidenced by limited worm burden reduction.

**Fig 2:**
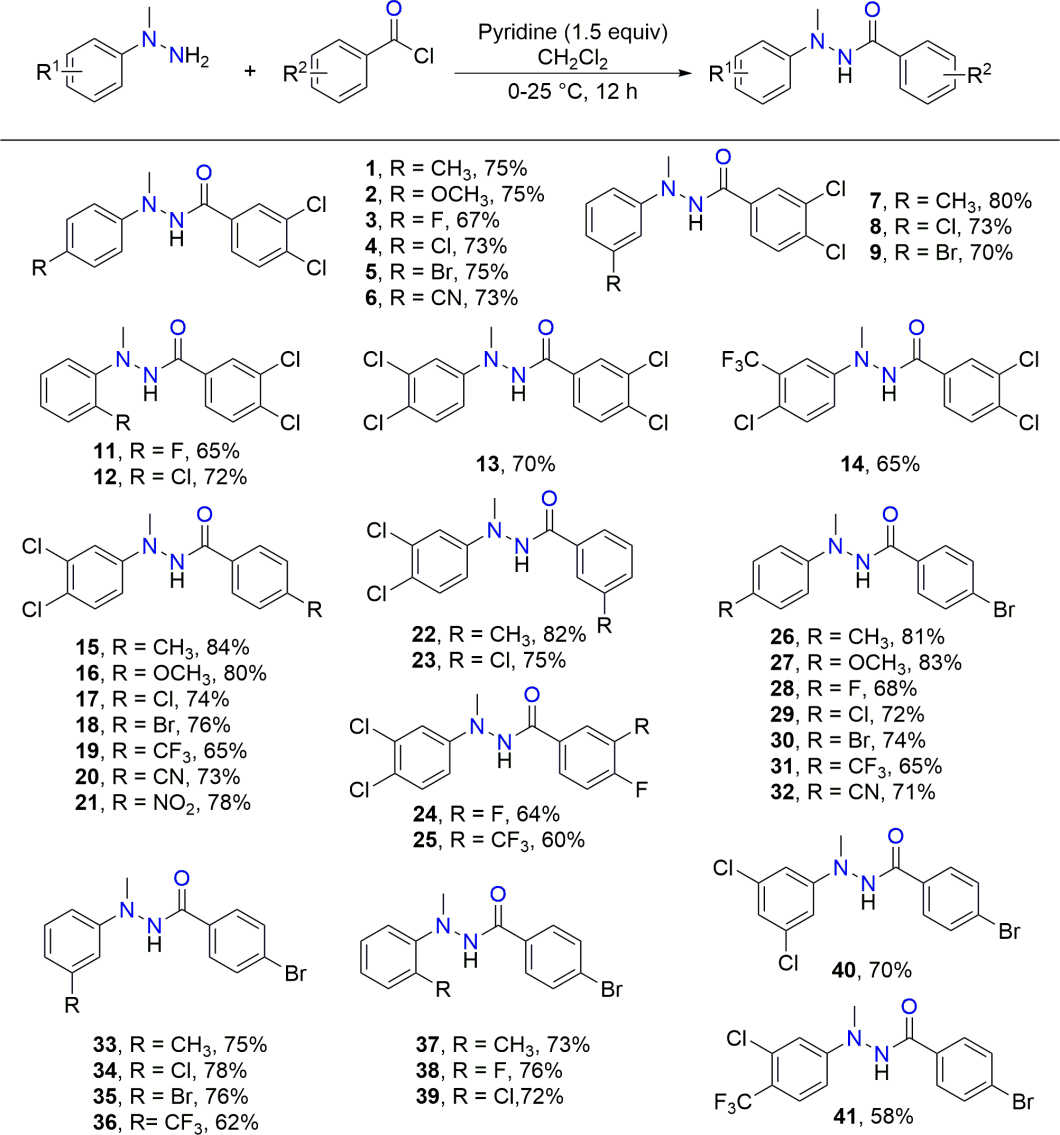
Synthesis of *N*-aryl-*N*′-methylbenzohydrazides (1–41).

The present study established a systematic structure-activity relationship (SAR) analysis of *N*,*N*′-diarylurea derivatives exhibiting *Schistosoma japonicum* inhibition (24). Using a rational drug design framework, we first replaced the urea linker with a hydrazine isosteric moiety to enhance metabolic stability (25). Subsequently, we undertook comprehensive structural diversification, systematically optimizing electron-donating and electron-withdrawing substituents at *ortho*-, *meta*-, and *para*-positions of both aromatic rings. This strategy culminated in synthesizing 41 *N*-aryl-*N*′-methylbenzohydrazide analogs (Fig. 2). We rigorously evaluated all compounds for dual-stage antischistosomal efficacy using standardized *in vitro* adult motility assays, and then *in vivo* worm burden reduction studies in ICR mice for the selected compounds were investigated. Additionally, we assessed hepatotoxicity and acute oral toxicity of selected compounds in murine models according to established methods (26).

## RESULTS

### Chemistry

Although various synthetic routes for substituted benzohydrazides have been reported (16), the synthesis of *N*-aryl-*N*′-methylbenzohydrazides (1–41) is detailed in Fig. 2 using a classical strategy. Briefly, these compounds were prepared via nucleophilic acyl substitution between *N*-aryl-*N*′-methylhydrazines and substituted benzoyl chlorides in the presence of pyridine at 0°C–25°C (room temperature) for 12 h. The target products were isolated in 58%–84% yields and characterized by infrared (IR), nuclear magnetic resonance (NMR), and high-resolution mass spectrometry (HRMS).

### *In vitro* antischistosomal activity

Bioassays revealed dose-dependent antischistosomal efficacy in most synthesized compounds within 72 h post-treatment (Table 1). Notably, seven lead candidates (1, 3, 5, 8, 9, 13, and 14) exhibited >80% adult worm mortality at 72 h, with compound 5 showing exceptional time-dependent potency (95% clearance within 48 h).

**TABLE 1:** *In vitro* effects of compounds (1–41) against adult worms of *S. japonicum* (Chinese strain) at 100 μM*^a^*^,^*^b^*^,^*^c^*

Compound	No. of adult worms	Mortality (%)			
		24 h	36 h	48 h	72 h
Negative control	10	0	0	0	0
1	10	0	5	60	100
2	10	0	0	0	0
3	10	0	0	45	85
4	10	0	0	0	10
5	10	35	65	95	100
6	10	0	0	0	20
7	10	0	0	0	0
8	10	0	0	40	100
9	10	0	0	15	85
10	10	0	0	0	20
11	10	0	0	0	0
12	10	0	0	0	0
13	10	0	15	50	100
14	10	15	25	55	95
15	10	0	20	35	65
16	10	0	0	65	100
17	10	0	5	25	70
18	10	10	50	80	100
19	10	0	0	35	80
20	10	0	0	60	100
21	10	0	0	30	65
22	10	0	0	0	70
23	10	0	5	40	95
24	10	0	5	45	90
25	10	0	0	30	80
26	10	0	0	10	10
27	10	0	0	0	0
28	10	0	0	0	0
29	10	0	0	0	35
30	10	0	10	65	100
31	10	0	10	55	75
32	10	0	0	0	0
33	10	0	15	40	70
34	10	0	0	0	90
35	10	0	0	30	60
36	10	0	0	10	80
37	10	0	0	0	0
38	10	0	0	10	10
39	10	0	0	10	20
40	10	0	20	70	100
41	10	35	70	80	100
MMV665852	10	100	100	100	100
PZQ	10	100	100	100	100

^*a*^ Negative control group: 2 µL dimethyl sulfoxide (DMSO).

^*b*^ Values correspond to the sum of the adult schistosomes obtained from three separate experiments performed in triplicate (*n* = 2) and quadruplicate (*n* = 1).

^*c*^ Significant difference compared with untreated control group (two-way ANOVA, *P* < 0.05).

Following systematic investigation of Ring A substituent effects, we maintained chlorine substituents at 3,4-positions of Ring A as the optimized configuration and explored Ring B modifications. SAR analysis revealed potent antiparasitic efficacy across all compounds within 72 h. Among the tested compounds, compounds 16, 18, 20, 23, and 24 demonstrated >90% worm mortality at 72 h, with compound 18 exhibiting superior time-dependent efficacy (80% mortality at 48 h).

With bromine on Ring B established as optimal, we refined Ring A modifications. As shown in Table 1, the lead compounds (30, 40, and 41) exhibited remarkable parasiticidal activity, achieving complete adult worm eradication (100% mortality) within 72 h of exposure. Compound 41 emerged as the most potent analog, demonstrating a rapid onset of action with 70% mortality observed at the 36 h mark. Electron-withdrawing groups (-Cl, -Br, -NO_2_, -CF_3_, -CN) on Ring A enhanced potency relative to electron-donating analogs.

We then determined the LC_50_ values for compounds 5, 18, and 41, which showed good *in vitro* activity against adult *S. japonicum* (Table 2). The results demonstrated that compound 41 had the lowest LC_50_ and highest activity. Compounds 5 and 18 also showed significant activity comparable to PZQ (positive control).

**TABLE 2:** LC_50_ (72 h) values of compounds (5, 18, and 41) against adult worms of *S. japonicum* (Chinese strain) *in vitro*

Compound	LC_50_ (μM)	Compound	LC_50_ (μM)
5	80.20 ± 1.80	MMV665852	12.54 ± 2.36
18	83.00 ± 1.90	PZQ	43.76 ± 8.26
41	61.40 ± 1.80		

**Fig 3:**
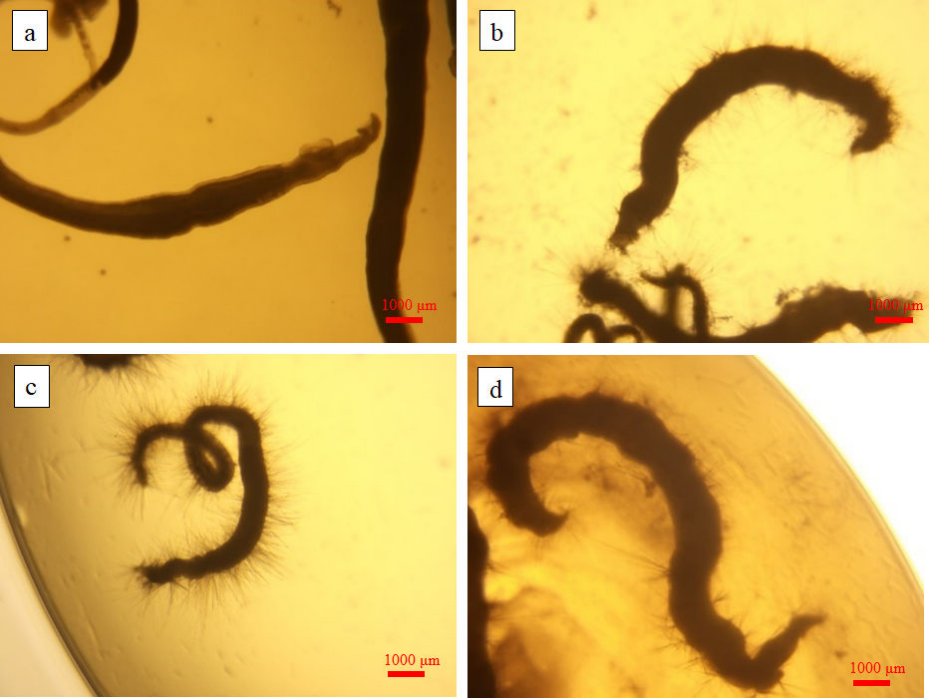
The optical microscopy images of adult worms of *S. japonicum* treated with 100 µM compouds 5, 18, and 41. (**a**) Negative control group for 72 h; (**b**) compound 5 for 72 h; (**c**) compound 18 for 72 h; (**d**) compound 41 for 72 h.

Optical microscopy of drug-treated *S. japonicum* (100 µM for 72 h) revealed pathognomonic morphological changes (Fig. 3). Negative controls maintained tegumental transparency with intact suckers and muscular relaxation. Treated parasites exhibited (i) significant somatic edema and cuticular curvature; (ii) progressive integumental hyperpigmentation; (iii) complete immobilization with 100% mortality. In addition, crystalline egesta (needle-like particulates, 50–150 µm) emanating from the tegument within 72 h were likely precipitated drug metabolites—potentially attributable to limited aqueous solubility in RPMI 1640 medium and phase separation of hydrophobic derivatives during transmembrane transport.

### Scanning electron microscope examination *in vitro*

**Fig 4:**
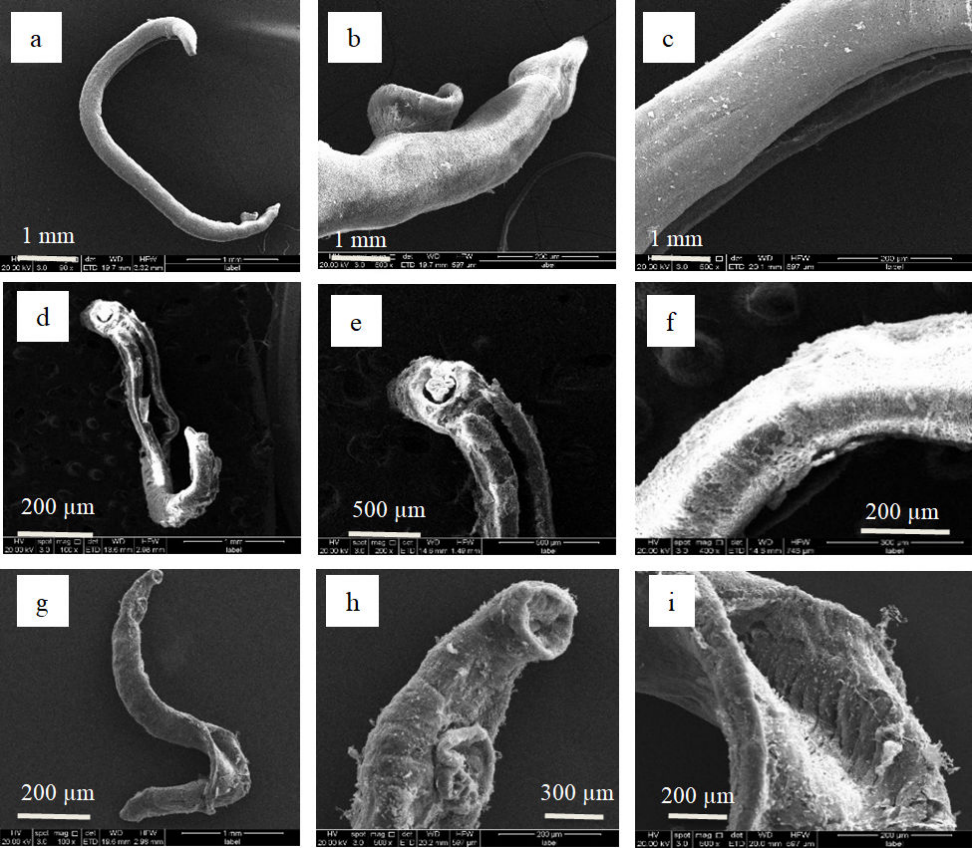
Scanning electron micrographs of adult male *S*. *japonicum* worms. Negative control group (0.1% DMSO in RPMI 1640 medium): (**a**) the entire male worm, (**b**) the male worm abdominal sucker, (**c**) the male worm body. Positive control group (treated with 100 μM PZQ): (d) the entire male worm, (**e**) the male worm abdominal sucker, (**f**) the male worm body. Experimental group (treated with 100 μM compound 5): (**g**) the entire male worm, (**h**) the male worm abdominal sucker, (**i**) the male worm body.

**Fig 5:**
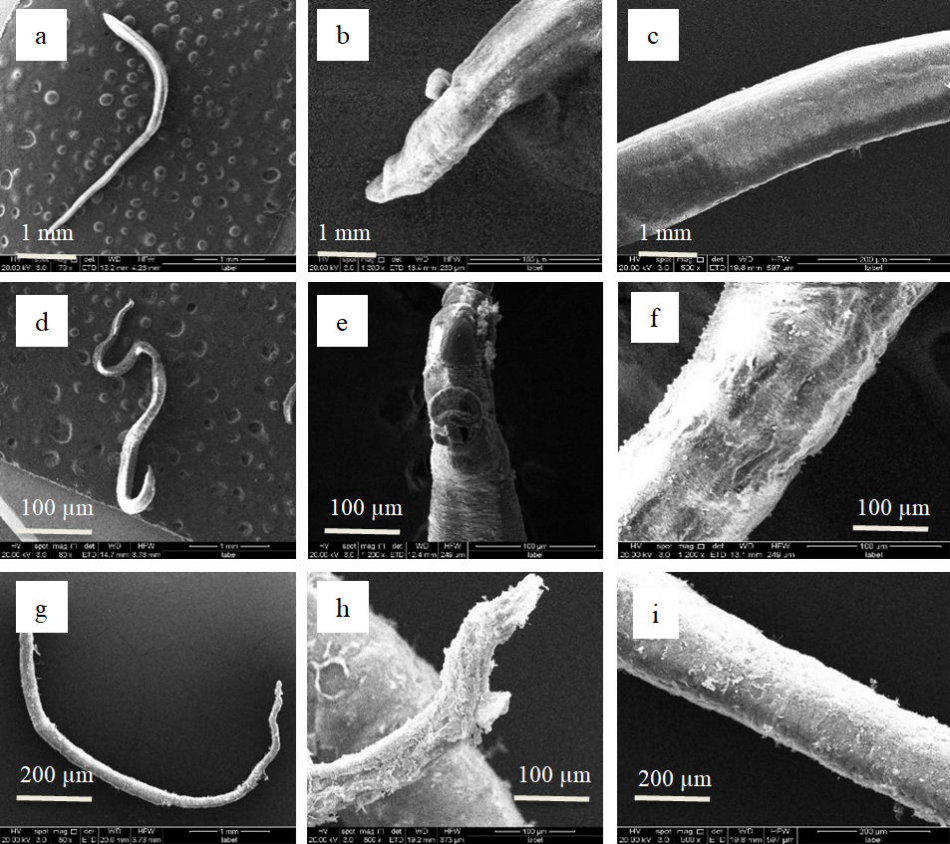
Scanning electron micrographs of adult female *S*. *japonicum* worms. Negative control group (0.1% DMSO in RPMI 1640 medium): (**a**) the entire female worm, (**b**) the female worm’s abdominal sucker, (**c**) the female worm’s body. Positive control group (treated with 100 μM PZQ): (**d**) the entire female worm, (**e**) the female worm’s abdominal sucker, (**f**) the female worm’s body. Experimental group (treated with 100 μM compound 5): (**g**) the entire female worm, (**h**) the female worm’s abdominal sucker, (**i**) the female worm’s body.

Ultrastructural characterization of drug-induced tegumental damage in adult *S. japonicum* was performed using scanning electron microscopy (SEM) to systematically evaluate morphological perturbations (Fig. 4 and 5). Comparative analysis revealed distinct pathomorphological features across groups. In negative controls (Fig. 4a through c and 5a through c), both male and female adult worms maintained structural integrity with continuous tegumental surfaces, well-defined oral/ventral suckers exhibiting characteristic muscular organization, preserved tubercular morphology with intact apical spines, uniformly distributed sensory floral papillae, and no muscular layer exposure or subtegumental disruption. However, in the praziquantel (PZQ)-treated group (Fig. 4d through f and 5d through f), male adult worms showed extensive tegumental desquamation, complete muscular layer exposure with striation disruption, and ventral sucker collapse. Additionally, female worms also displayed multifocal vesiculation, ulcerative lesions penetrating the basal lamina, and progressive tegumental corrugation.

In the compound 5-treated group (Fig. 4g through i and 5g through i), male adult worms exhibited complete spine ablation in gynecophoric canals, suctorial constriction, and muscular layer protrusion through disrupted tegument. Female adult worms showed full-thickness sucker ulceration with stromal collapse, atrophic transformation with coalescing vesicles, and advanced tissue disintegration with tegumental wrinkling. These compound 5-specific ultrastructural alterations demonstrate significantly enhanced tegumental disintegration relative to controls.

### *In vivo* antischistosomal activity

Subsequently, we evaluated the *in vivo* antischistosomal efficacy of compounds 5, 18, and 41 through oral administration at a dosage of 100 mg/kg/day administered consecutively for 5 days. As summarized in Table 3, all three compounds demonstrated measurable antiparasitic activity against *S. japonicum* worms across different developmental stages.

**TABLE 3:** The effects of oral administration of compounds 5, 18**,** and 41 on *S*. *japonicum* (Chinese strain) in mice *in vivo* at a dose of 100 mg/kg/day for 5 days^*a*^*^,b^*

Compound	Number of worms		Worm burden reduction (WBR)	
	Juvenile	Adult	Juvenile	Adult
5	30	29	25%	34%
18	24	35	40%	20%
41	28	31	30%	30%
MMV665852	32	36	20%	10%
PZQ	39	0	0%	100%
Negative control	40	44	0%	0%

^*a*^ Values correspond to the sum of the adult schistosomes obtained from three separate experiments performed in triplicate (*n* = 2) and quadruplicate (*n* = 1).

^*b*^ Significant difference compared with untreated control group (two-way ANOVA, *P* < 0.05).

### Hepatotoxicity

Following maturation, Schistosoma parasites initiate oviposition, with a proportion of eggs migrating via the circulatory system to hepatic tissues. This ectopic deposition induces granuloma formation that subsequently elicits a cascade of immune-mediated pathologies, ultimately compromising hepatic and renal functions in murine models. To evaluate therapeutic efficacy, retro-orbital blood sampling was performed for comprehensive hepatic function profiling, enabling systematic assessment of pharmacological impacts on schistosomiasis-associated organ damage. As shown in Table 4, juvenile-stage pharmacological interventions revealed distinct compound-specific effects. For bilirubin metabolism, experimental groups receiving compounds 18 and 41 exhibited significantly reduced total bilirubin (TBIL) levels compared to negative controls, indicative of hepatoprotective efficacy against schistosome-induced hepatic inflammation. In this case, alanine aminotransferase (ALT) and aspartate aminotransferase (AST) levels remained comparable across all treatment groups and negative controls, suggesting that these compounds did not exacerbate liver damage. Notably, gamma-glutamyltransferase (GGT) activity in compounds 5 and 18 treatment groups showed marked reduction, approaching baseline levels observed in uninfected controls, confirming functional hepatic recovery. Blood urea nitrogen (BUN) measurements showed compound 18 administration induced significant elevation, suggesting nephrotoxic potential, whereas compounds 5 and 41 maintained BUN levels within physiological ranges.

**TABLE 4:** Serum levels of TBIL, ALT, AST, and BUN from mice infected with schistosomiasis after treatment with compounds 5, 18**,** and 41 in juvenile stages^*a*^

Item	Compound 5	Compound 18	Compound 41	Negative control group	Normal control group
TBIL (1.7–15.4 µM)	7.48 ± 1.64	2.98 ± 1.03	3.61 ± 1.39	3.74	3.59
ALT (28–132 U/L)	357.67 ± 176.92	546.00 ± 82.15	364.00 ± 329.78	385.00	35.00
AST (59–247 U/L)	602.33 ± 309.33	673.67 ± 153.58	557.67 ± 630.32	884.00	128.00
GGT (1–15 U/L)	0.73 ± 0.06	0.93 ± 0.81	1.60 ± 0.17	2.00	0.60
BUN (4.9–10.4 mM)	9.83 ± 1.43	12.53 ± 1.31	6.18 ± 4.85	9.20	6.38

^*a*^ TBIL, total bilirubin; ALT, alanine transaminase; AST, aspartate transaminase; GGT, gamma-glutamyltransferase; BUN, blood urea nitrogen.

In addition, adult-stage infection models presented distinct pathophysiological responses. As shown in Table 5, TBIL levels exhibited paradoxical elevation across all treatment groups, potentially associated with post-phlebotomy hemolytic artifacts. Compared to the negative control, the ALT and AST enzyme levels for compounds 5, 18, and 41 decreased, indicating that these three compounds have a certain alleviating effect on liver function damage caused by *S. japonicum* worms in mice. The GGT levels for compounds 5 and 41 were lower than those in the negative control group, suggesting that these two compounds provide greater protection to the liver. Additionally, the BUN levels for compounds 5, 18, and 41 were lower than those in the negative control group, indicating that these three compounds also have a certain alleviating effect on kidney function damage caused by *S. japonicum* worms in mice.

**TABLE 5:** Serum levels of TBIL, ALT, AST, and BUN from mice infected with schistosomiasis after treatment with compounds 5, 18**,** and 41 in adult stages^*a*^

Item	Compound 5	Compound 18	Compound 41	Negative control group	Normal control group
TBIL (1.7–15.4 µM)	6.70 ± 3.39	18.73 ± 6.05	7.95 ± 2.11	3.74	3.59
ALT (28–132 U/L)	313.33 ± 166.21	322.33 ± 239.58	274.67 ± 186.53	385.00	35.00
AST (59–247 U/L)	527.33 ± 303.66	783.67 ± 457.47	453.33 ± 217.99	884.00	128.00
GGT (1–15 U/L)	0.73 ± 0.40	6.73 ± 9.70	0.87 ± 0.32	2.00	0.60
BUN (4.9–10.4 mM)	8.80 ± 0.96	8.68 ± 1.22	7.79 ± 2.11	9.20	6.38

^*a*^ TBIL, total bilirubin; ALT, alanine transaminase; AST, aspartate transaminase; GGT, gamma-glutamyltransferase; BUN, blood urea nitrogen.

### Acute oral toxicity and cytotoxic screening

Systematic toxicological evaluation of compounds 5, 18, and 41 in ICR mice revealed favorable safety profiles. Acute oral LD_50_ values were determined to be 1,395 mg/kg (Table 6). According to standard toxicity classification (27), this value (300–2,000 mg/kg) falls within the low toxicity range. However, compounds 5, 18, and 41 showed cytotoxic effects against the MCF-7 (human breast cancer) and HepG2 (human liver cancer) cell lines (Table 7).

**TABLE 6:** The values of LD_50_ of compounds 5, 18**,** and 41 on the healthy mice

Compound	LD_50_ (mg/kg)	LogLD_50_
5	>2,000	>3.30
18	>2,000	>3.30
41	>2,000	>3.30

**TABLE 7:** IC_50_ (μM) values of selected compounds on tumor cells (*in vitro*)

Compound	MCF-7	HepG2
5	43.78 ± 2.53	23.24 ± 1.43
18	40.87 ± 2.82	22.64 ± 1.48
41	39.24 ± 3.53	20.69 ± 1.36
Cisplatin	1.17 ± 0.13	3.51 ± 0.14

## DISCUSSION

During initial structural optimization, chlorine substituents at C3 and C4 positions of the B-ring were preserved to maintain a conserved pharmacophore, while systematic A-ring modifications were evaluated for their effects on *in vitro* schistosomicidal activity against adult *S. japonicum* (Fig. 2, Table 1). SAR analysis of A-ring modifications revealed three critical principles: (i) C2 position sensitivity: substituents at C2 (compounds 10–12) displayed markedly reduced bioactivity regardless of electronic properties, suggesting steric hindrance; (ii) C3 electronic effects: electron-withdrawing groups (-Cl, -Br) at C3 (compounds 8–9) significantly enhanced efficacy; (iii) C4 halogen dominance: halogenation at C4 proved pivotal, with brominated derivative 5 achieving optimal efficacy (100% mortality at 72 h). These findings underscore the critical role of A-ring C4 halogen substituents in mediating antischistosomal activity through putative electronic and steric interactions.

As shown in Table 1, the experimental results showed that electron-withdrawing groups (-F, -Cl, -Br, -CF_3_, -CN) on Ring B significantly enhanced activity, with bromine substitution yielding optimal bioactivity—suggesting potential halogen bonding or enhanced electronic effects in the receptor environment. Based on these findings, we propose the following SAR for *in vitro* antischistosomal activity of *N*-aryl-*N*′-methylbenzohydrazides: (i) with 3,4-Cl on Ring B: halogenation (Cl/Br) at Ring A-C3 enhances efficacy; Ring A-C4 substitution with F, Br, or CH_3_ improves activity; synergistic enhancement occurs with dual electron-withdrawing groups (EWGs) at Ring A-C3/C4. (ii) With 3,4-Cl on Ring A: EWGs at Ring B-C3 substantially increase activity; both electron-donating groups and EWGs at Ring B-C4 enhance efficacy; optimal activity with dual EWGs at Ring B-C3/C4.

SEM unveiled critical ultrastructural pathologies in drug-exposed (compound 5) parasites, characterized by widespread tegumental erosion with ulcerative lesions, denudation of subtegumental musculature, contractile collapse of oral/ventral suckers, and pronounced cuticular corrugation in male and female specimens (Fig. 4 and 5).

*In vivo* experiments, quantitative analysis revealed stage-dependent variations in therapeutic effectiveness: compound 5 exhibited 25% and 34% worm burden reduction rates in juvenile (schistosomula) and adult stages, respectively (Table 3). Compound 18 displayed differential efficacy with a 40% reduction in the juvenile stage compared to a 20% in the adult stage. Compound 41 also maintained consistent activity, achieving 30% parasite clearance in both developmental phases. Notably, comparative evaluation identified compound 18 as the most potent agent against *S. japonicum* worms in the juvenile stage (40% reduction rate), while compound 5 showed superior efficacy against adult parasites (34% reduction rate). This stage-specific pharmacological profile suggests distinct mechanisms of action may underlie the observed antiparasitic effects. While PZQ demonstrates therapeutic efficacy in the adult stage (100% worm burden reduction), it exhibits complete pharmacological inertness during the schistosomula stage, showing no measurable anthelmintic activity (0% parasite clearance) against juvenile worms.

**Fig 6:**
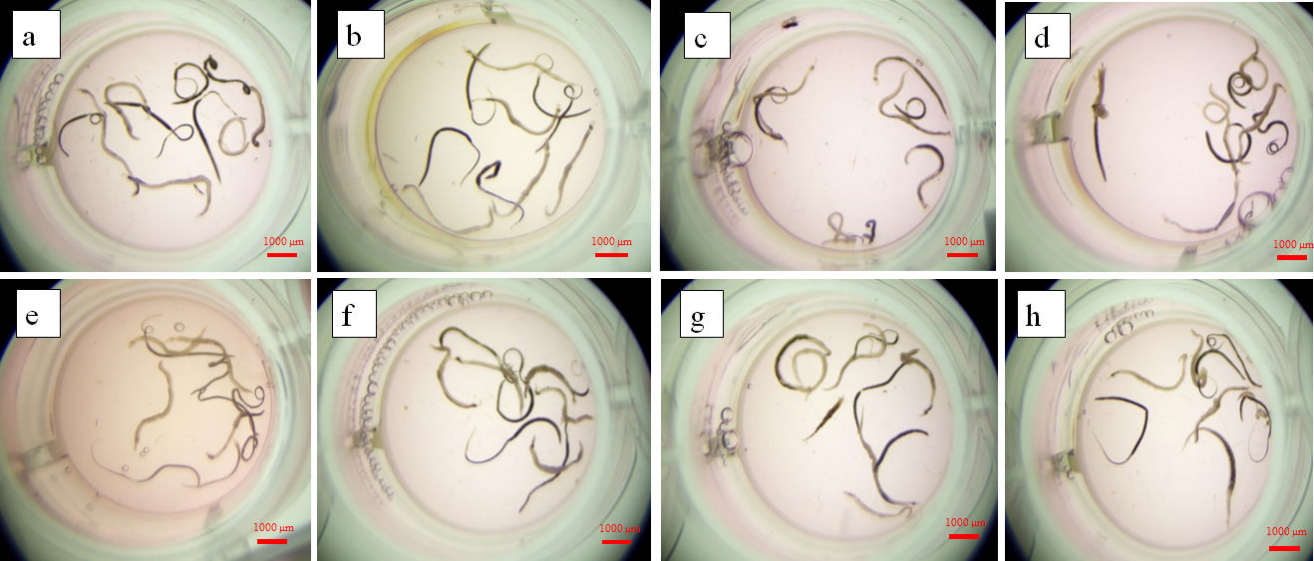
The morphology of the worms after treatment with compounds 5, 18, and 41 *in vivo*. (**a**) Negative control group in the juvenile stage; (**b**) treatment with PZQ in the juvenile stage; (**c**) treatment with compound 5 in the juvenile stage; (**d**) treatment with compound 18 in the juvenile stage; (**e**) treatment with compound 41 in the juvenile stage; (**f**) treatment with compound 5 in the adult stage; (**g**) treatment with compound 18 in the adult stage; (**h**) treatment with compound 41 in the adult stage.

The morphology of parasites was observed following therapeutic intervention with compounds 5, 18, and 41 across both developmental stages of *S*. *japonicum in vivo*. As shown in Fig. 6, in the juvenile stage experiments, praziquantel-treated positive controls showed no significant tegumental alterations compared to negative controls, confirming its limited chemotherapeutic impact on juvenile forms (Fig. 6a and b). In contrast, experimental groups (treatment with compounds 5, 18, and 41) exhibited marked stunting of male schistosomes, with mean length reductions, indicating substantial disruption of normal developmental progression (Fig. 6c, d, and e). The adult-stage morphological analysis revealed significant drug-induced modifications (Fig. 6f, g, and h). Compounds 5, 18, and 41 significantly affect the development of *S. japonicum* in the adult stage after administration, making the males thicker and shorter.

To demonstrate measurable hepatorenal protective effects, biochemical assessments were further investigated (Tables 4 and 5). These findings systematically demonstrate that juvenile-stage chemotherapeutic intervention with test compounds effectively mitigates schistosomiasis-induced multiorgan dysfunction through distinct mechanisms: compounds 5 and 41 confer dual hepato-renal protection, while compound 18 exhibits hepatoprotective capacity, accompanied by possible nephrotoxic sequelae.

**Fig 7:**
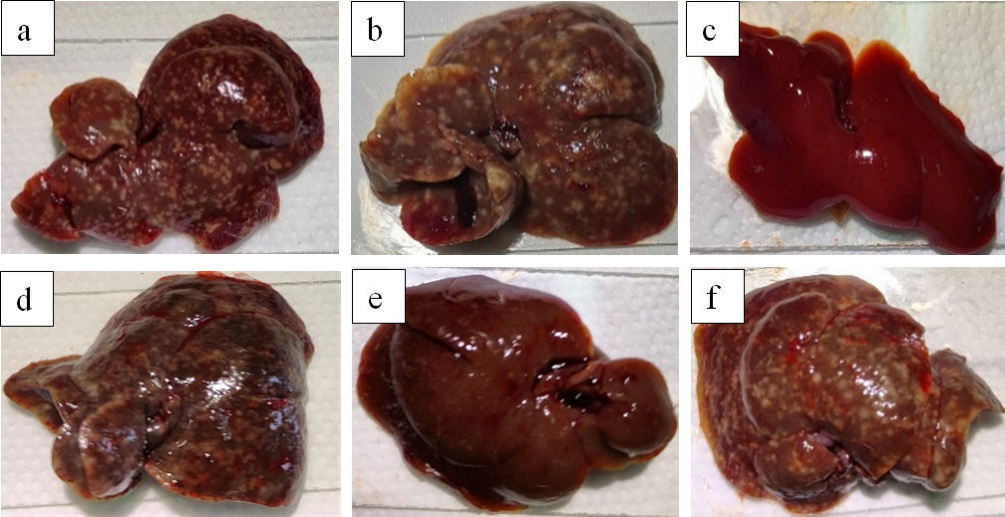
The optical microscopy images of livers of mice after treatment with compounds 5, 18, and 41 in the juvenile stage *in vivo*. (**a**) Negative control group; (**b**) treatment with PZQ; (**c**) normal control group; (**d**) treatment with compound 5; (**e**) treatment with compound 18; (**f**) treatment with compound 41.

Following pharmacological intervention at the juvenile worm stage, murine specimens were euthanized at 45 days post-infection (dpi) for hepatic examination (Fig. 7). Untreated negative controls demonstrated extensive oviposition within hepatic tissues, with parenchymal coloration comparable to uninfected specimens. The praziquantel-treated positive control cohort exhibited a darker hepatic coloration relative to negative controls, though without statistically significant ovicidal efficacy. These findings suggest limited antischistosomal activity and marginal hepatotoxic effects of praziquantel when administered during juvenile schistosomal stages. Notably, mice administered compound 18 during juvenile worm development displayed both significantly reduced egg burdens and near-physiological hepatic pigmentation compared to compounds 5 and 41. This evidences compound 18’s superior larvicidal efficacy and hepatoprotective properties *in vivo*. Conversely, compounds 5 and 41 showed lower parasiticidal activity during juvenile stages while inducing marked hepatic discoloration, indicative of reduced therapeutic potential.

**Fig 8:**
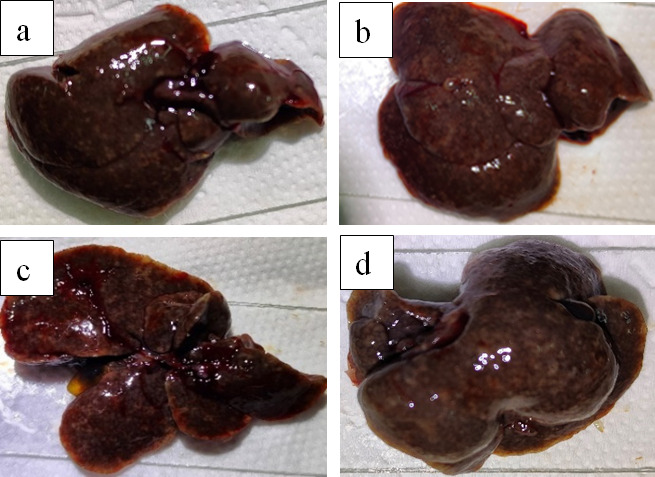
The optical microscopy images of livers of mice after treatment with compounds 5, 18, and 41 in the adult stage *in vivo*. (**a**) Treatment with PZQ; (**b**) treatment with compound 5; (**c**) treatment with compound 18; (**d**) treatment with compound 41.

For adult worm stage therapeutic evaluation, hepatic analyses conducted at 45 dpi revealed distinct pharmacological profiles (Fig. 8). Praziquantel administration during adult worm maturation significantly reduced both ovum dimensions and egg counts versus negative controls, confirming its adulticidal efficacy. However, observed hepatic chromatic alterations suggest concurrent drug-induced hepatotoxicity. In the experimental group, compound 5 was the most effective drug for killing adult worms, while maintaining normal hepatic pigmentation—indicative of enhanced hepatic preservation. Compounds 18 and 41 showed intermediate toxicological profiles, with hepatic coloration significantly lighter than praziquantel-treated specimens yet comparable egg burdens to untreated controls, suggesting reduced parasiticidal potency with diminished hepatotoxic effects relative to standard therapy.

In conclusion, we strategically designed and synthesized a series of *N*-aryl-*N*′-methylbenzohydrazides to identify novel therapeutics targeting *S. japonicum*. Through comprehensive *in vitro* screening, compounds 5, 18, and 41 emerged as lead candidates with pronounced anthelmintic activity. *In vivo* pharmacological profiling revealed distinct developmental-stage efficacy: compound 18 achieved superior juvenile-stage schistosomicidal effects, while compound 5 demonstrated optimal adulticidal potency. Collectively, we propose that *N*-aryl-*N*′-methylbenzohydrazides induce catastrophic failure of the schistosomal integumentary system, disrupting essential biological processes, including transtegumental nutrient trafficking, osmoregulatory homeostasis, and immune evasion mechanisms. This tegumental compromise likely facilitates direct pharmacological penetration, overriding the parasite’s primary defenses. Biochemical assessments further demonstrated measurable hepatorenal protective effects across the compound series. These findings establish *N*-aryl-*N*′-methylbenzohydrazides as a highly promising chemotype for antischistosomal drug development. Future studies will delineate structure-activity relationships and molecular targets through integrated transcriptomic and proteomic approaches.

## MATERIALS AND METHODS

### General

Melting points were determined using a Büchi M-565 digital melting point apparatus (uncorrected values). IR spectra were recorded on a Thermo Scientific Nicolet iS50 spectrometer using KBr pellets (ν in cm^−1^). ^1^H and ^13^C{^1^H} NMR spectra were acquired on a Bruker Avance 400 spectrometer (400 MHz) using dimethyl sulfoxide-*d*_6_ (DMSO-*d*_6_) as the solvent and tetramethylsilane as the internal standard. HRMS data were obtained on a Shimadzu LCMS-9030 instrument (LCMS-IT-TOF). Thin-layer chromatography (TLC) and column chromatography were performed using commercially available 100–400 mesh silica gel. All reagents were purchased from Innochem (Beijing) Technology Co., Ltd., and used without further purification unless otherwise specified.

### General procedure for the preparation of compounds 1–41

To a solution of methylhydrazine derivatives (0.10 mmol) in anhydrous dichloromethane (1.0 mL) under N_2_ at 0°C (ice-water bath), pyridine (0.15 mmol) was added via syringe pump over 5 min. Benzoyl chloride (0.10 mmol) dissolved in anhydrous dichloromethane (1.0 mL) was then added dropwise through a pressure-equalizing dropping funnel over 30 min with vigorous stirring (800 rpm). The reaction mixture was warmed gradually to ambient temperature (25°C ± 2°C) and stirred for 12 h (monitored by TLC, EtOAc/hexane 5:1). After completion, the mixture was quenched with water, extracted with ethyl acetate, and dried over anhydrous Na_2_SO_4_. The solvent was removed under reduced pressure, and the residue was purified by silica gel chromatography (petroleum ether/ethyl acetate, vol/vol = 5:1) to afford products 1–41.

### Parasite and animal models

*S. japonicum* cercariae (Chinese strain) were acquired from infected *Oncomelania hupensis* snails (Wuxi Insect Vector Biology Co., Ltd., China) (28). ICR mice (6–8 weeks, 22–25 g; Hunan SJA Laboratory Animal Co., Ltd., China) were percutaneously infected via abdominal exposure to 60 ± 5 cercariae. Adult schistosomes were perfused from the hepatic portal system at 35 dpi using a modified Duvall and DeWitt method (29). Animals were housed under controlled conditions (22°C ± 1°C, 12 h light/dark cycle) with *ad libitum* access to feed and water.

### *In vitro* antischistosomal assay

Adult *S. japonicum* worms isolated from ICR mice were maintained in sterile culture medium (2.0 mL) comprising 77% RPMI 1640 (Lanzhou Bailing Biotech Co., Ltd.; pH 7.5, HEPES-buffered), 20% heat-inactivated fetal bovine serum (Lanzhou Minhai Bio-Engineering Co., Ltd.), and 3% penicillin-streptomycin (100×; 10,000 U/mL penicillin, 10,000 μg/mL streptomycin; New Cell & Molecular Biotech Co., Ltd., China). Incubation was performed at 37°C under 5% CO_2_. Test compounds were dissolved in DMSO as 0.1 M stock solutions; 2.0 μL aliquots were added to worm-containing media (final concentration: 100 μM in 0.1% DMSO). Negative controls received equivalent volumes of vehicle (0.1% DMSO in RPMI 1640). Parasite viability (motor activity, tegumental integrity, survival) was assessed every 12 h for 72 h using an Olympus CKX53 inverted microscope (*n* ≥ 3 independent replicates).

### SEM examination *in vitro*

Adult *S. japonicum* worms (male/female) underwent standardized fixation after *in vitro* exposure to compounds 5, 18, and 41 over a period of 12 h. First, the samples were immersed in 2.5% glutaraldehyde (prepared with 40 mL 0.2 M phosphate-buffered saline [PBS, pH 7.4], 5 mL 50% glutaraldehyde stock, and 55 mL ultrapure water) at 4°C for 24 h. After triple washes in PBS for 5 min each, worms were rinsed with deionized water for 10 min. The next dehydration sequence included ethanol gradient (50%, 70%, 80%, 90%, 100%; 5 min/step) and *tert*-butanol gradient (50%, 70%, 90%, 100%; 10 min/step). The worms were freeze-dried, directly sputter-covered with a thin gold layer using a Sputter Coater, and imaged using a scanning electron microscope (Quanta 450).

### *In vivo* antischistosomal assay

Lead compounds 5, 18, and 41 (100 mg/kg) were orally administered once daily for 5 consecutive days to infected mice (*n* = 6/group) during two therapeutic windows: (i) Juvenile stage: treatment initiated at 9 dpi, terminal sacrifice at 45 dpi; (ii) adult stage: treatment initiated at 35 dpi, terminal sacrifice at 45 dpi. Positive controls received praziquantel (PZQ, 100 mg/kg/day ×5 days); negative controls received PBS (0.3 mL). Mice treated at 9 dpi were euthanized 36 days post-treatment; those treated at 35 dpi were euthanized 10 days post-treatment. Adult worms were perfused from the hepatic portal system and mesenteric veins. Parasite burden was quantified by worm enumeration, and worm burden reduction (WBR) was calculated:


WBR (%)=[1−Mean worm count (treated) / Mean worm count (control)]×100% .

### Acute oral toxicity

ICR mice (*n* = 10/group), aged 6 to 8 weeks, received a single oral dose of compounds 5, 18, and 41 (732–2,234 mg/kg). LD_50_ values were determined by Probit analysis (SPSS 26.0).

### Cytotoxicity assay

The cytotoxicity of compounds 5, 18, and 41 against human liver cancer (HepG2) and human breast cancer (MCF-7) cell lines *in vitro* was evaluated using the MTT assay, with cisplatin as a positive control. Cells were plated into 96-well plates and incubated in 100 μL of Dulbecco’s modified Eagle’s medium for 24 h. Test compounds at specified final concentrations were then added to the culture medium. After 48 h incubation, MTT dye (10 μL, 0.5 mg/mL) was added, and cells were incubated for an additional 4 h at 37°C in 5% CO_2_. Formazan crystals were dissolved in 100 μL DMSO per well, and absorbance at 570 nm (OD_570_) was measured using an ELx800 microplate reader (Bio-Tek, Winooski, VT, USA) with background subtraction. Cytotoxicity was expressed as IC_50_ (the concentration causing 50% reduction in cell growth compared to untreated controls). Experiments were performed in triplicate, with results presented as mean ± standard deviation.

## Data Availability

Data will be made available on request.
